# Evolutionary Approach for Relative Gene Expression Algorithms

**DOI:** 10.1155/2014/593503

**Published:** 2014-03-23

**Authors:** Marcin Czajkowski, Marek Kretowski

**Affiliations:** Faculty of Computer Science, Bialystok University of Technology, Wiejska 45a, 15-351 Białystok, Poland

## Abstract

A Relative Expression Analysis (RXA) uses ordering relationships in a small collection of genes and is successfully applied to classiffication using microarray data. As checking all possible subsets of genes is computationally infeasible, the RXA algorithms require feature selection and multiple restrictive assumptions. Our main contribution is a specialized evolutionary algorithm (EA) for top-scoring pairs called EvoTSP which allows finding more advanced gene relations. We managed to unify the major variants of relative expression algorithms through EA and introduce weights to the top-scoring pairs. Experimental validation of EvoTSP on public available microarray datasets showed that the proposed solution significantly outperforms in terms of accuracy other relative expression algorithms and allows exploring much larger solution space.

## 1. Introduction

Extracting accurate and simple rules that exploit marker genes is crucial in understanding and identifying casual relationships between specific genes. Finding a meaningful and robust classification rule is a real challenge; especially when in different studies of the same cancer, diverse genes are considered to be marked [1, 2].

A Relative Expression Analysis (RXA) was firstly proposed by Geman et al. in [3] and represents simple yet powerful set of classifiers. It is based on the relative orderings among the expressions of a small number of genes. Instead of using expression values directly, only ranks of the expression data are used, making the algorithms insensitive to data normalization procedures. Moreover, use of the ordering relationships for a small collection of genes has potential for identification of gene-gene interactions with plausible biological interpretation and direct clinical applicability [4]. Major and well-known drawback of RXA is a high computational complexity, which grows exponentially with the size of the collection of genes.

In this paper, we propose an Evolutionary Top-Scoring Pairs (EvoTSP) solution that combines the power of evolutionary approach with simplicity of relative expression algorithms. We managed to unify different top-scoring extensions, limit their restrictions, and with application of EA explore larger solution space. We have also changed the unweighted TSP voting, by introducing the weights of each gene pair.

The rest of the paper is organized as follows. In the next section the relative expression algorithms are briefly recalled.  Section 3 describes our motivation and Section 4 presents in detail the EvoTSP solution. Next, experimental validation on real-life microarray datasets is performed. The paper is concluded in the last section where possible future works are also sketched.

## 2. Background

The first and the most popular solution from RXA is called Top-Scoring Pair (TSP) [3]. It is based on pairwise comparisons of gene expression values. Discrimination between two classes depends on finding one pair of genes that achieves the highest ranking value called “score.”

Consider a gene expression microarray dataset consisting of *P* genes and *M* samples. Let the data be represented as a *P* × *M* matrix in which an expression value of *i*th gene from *j*th sample is denoted as *x*_*ij*_. Each row represents observation of a particular gene over *M* training samples, and each column represents a gene expression instance composed from *P* genes. Let us for the simplicity of presentation assume that there are only two classes, *C*_1_ and *C*_2_, and instances with indexes from 1 to *M*_1_ (*M*_1_ < *M*) that belong to the first class (*C*_1_) and instances from range 〈*M*_1_ + 1, *M*〉 to the second class (*C*_2_).

The TSP method focuses on gene pair matching (*i*, *j*) (*i*, *j* ∈ {1,…, *P*}, *i* ≠ *j*) for which there is the highest difference in probability *p* of an event *x*_*im*_ < *x*_*jm*_ (*m* = 1,2,…, *M*) between class *C*_1_ and *C*_2_. For each pair of genes (*i*, *j*) two probabilities are calculated, *p*_*ij*_(*C*_1_) and *p*_*ij*_(*C*_2_):

(1) $$\begin{array}{c} p_{ij}\left(C_{1}\right)=\frac{1}{\left|C_{1}\right|}\overset{M_{1}}{\underset{m=1}{\sum }}I\left(x_{im}<x_{jm}\right),\\ p_{ij}\left(C_{2}\right)=\frac{1}{\left|C_{2}\right|}\overset{M}{\underset{m=M_{1}+1}{\sum }}I\left(x_{im}<x_{jm}\right), \end{array}$$

where |*C*_1_| denotes the number of instances from class *C*_1_ and *I*(*x*_*im*_ < *x*_*jm*_) is the indicator function defined as

(2) $$\begin{array}{c} I\left(x_{im}<x_{jm}\right)=\{\begin{array}{cc} 1, & \text{if}\;x_{im}<x_{jm}\\ 0, & \text{if}\;x_{im}\geq x_{jm}. \end{array} \end{array}$$

TSP is a rank-based method; therefore, for each pair of genes (*i*, *j*) the “score” denoted Δ_*ij*_ is calculated as

(3) $$\begin{array}{c} \Delta_{ij}=\left|p_{ij}\left(C_{1}\right)-p_{ij}\left(C_{2}\right)\right|. \end{array}$$

In the next step, the algorithm chooses a pair with the highest score. There should be only one top pair in the TSP method; however, it is possible that multiple gene pairs achieve the same top score. In that case a secondary ranking proposed in [5] is used to eliminate draws. It is based on the rank differences in classes and samples.

In the literature, the TSP solution is extended in several directions, each having its pros and cons. In one of the first extensions called *k*-TSP [5] the number of top-scoring pairs included in the final prediction was increased. The classifier uses no more than *k* top scoring disjoint gene pairs that have the highest score. The parameter *k* is determined by the internal cross-validation and the simple majority vote is used to make the final decision.

Different approach for the TSP extension is discussed in [4] where authors instead of using several pairs of genes compare relationships for three genes. A three-gene version of RXA called Top-Scoring Triplet (TST) [4] was proposed as potentially more discriminating than TSP since there are six possible orderings that must be analyzed. With the TST solution authors successfully predict the germline BRCA1 mutations in breast cancer. This method was later extended in [6] where general idea of pairwise or triplet rank comparisons was proposed. The top-scoring N (TSN) algorithm uses generic permutations and dynamically adjusts the size to control both the permutation and combination space available for classification. Variable *N* denotes the size of the classifier; therefore, in case *N* = 2 the TSN algorithm simply reduces to the TSP method and when *N* = 3, the TSN can be seen as TST. The classifier's size can be defined by user or by internal cross-validation that checks classification accuracy for different values of *N* (on a training data, in a range specified by the user) and selects the classifier with the highest score.

A hybrid solution of *k*-TSP and a top-down induced decision tree is proposed in [7]. In each node of the decision tree called TSPDT a test analogous to the *k*-TSP method is searched. Then, the set of instances is divided according to decision of the best pair (or pairs) of genes in the current node and next; each derived subset goes to the corresponding branch. The process is recursively repeated for each branch until leaf node is reached. This solution was recently extended by global induction of decision tree called GTSPDT [8]. Preliminary experiments showed that this hierarchical evolutionary method can also be a good alternative to traditional relative expression algorithms.

**Figure 1:**
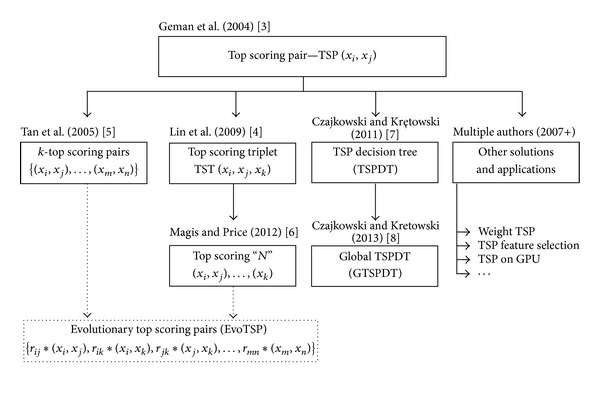
Evolution of the relative expression algorithms.

Figure 1 illustrates the extensions of the relative expression algorithms. We can observe that EvoTSP unifies two main extensions of the TSP solution: application of multiple pairs of genes instead of one and comparison relationships for more than two genes.

There exist other solutions in RXA like Weight  *k*-TSP [9] which focuses on the ratio of two genes in order to find more accurate top-scoring pairs. Different look at ranking the genes in microarray classification was also proposed in [10].

The RXA can be used as a feature selection in more complex classifiers [11–13] and as a protein expression classifier [14]. Multiple implementations of TSP-family solutions may be found as *R* package [15] or as a stand-alone application [16].

## 3. Motivation

The first drawback of RXA is the enormous computational requirement as the complexity of aforementioned algorithms is *O*(*k*∗*P* ^*N*^), where *k* is the number of top-scoring groups, *P* is the number of features, and *N* is the size of group of genes with which ordering relationships are compared. In the literature, there are some attempts of improving TSP performance by parallelization of the algorithm using graphic processing unit (GPU) for calculations [17]. Although the improvement is significant, the parameter *k* or/and *N* still must be small—the highest tested value of *N* equals 4 with *k* = 1 and only when *P* was significantly reduced by the feature selection. This illustrates how computationally demanding RXA is.

Finding accurate values of the parameters *k* and *N* is the second problem. The TSP extensions define them ad hoc or by internal cross-validation. The first way is strongly dependent on analyzed dataset and the second one is extremely time consuming and decreases the size of the training dataset which is usually very small in case of microarray data. In addition, it is not clear which extension should be preferred: *k*-TSP or TSN. It should be noted that the *k*-TSP algorithm cannot be replaced by the TST as *k*-TSP has restrictions to use only disjoint gene pairs. On the other side, the *k*-TST or *k*-TSN was not even analyzed in the literature, probably due to its computational complexity.

In this paper, we would like to limit aforementioned drawbacks of TSP extensions through the evolutionary approach. Our goal is to improve classification accuracy and identification of marker genes interactions. We let the EA to search for the best multiple pairwise comparisons of the gene expression values. The number of top-scoring pairs *k* is determined also by the evolution and with no restrictions on disjoint gene pairs; EvoTSP may compare relationships for more than two genes like in TSN. Application of EA to the RXA allows exploring larger solution space with reasonable computation time.

## 4. Evolutionary Top-Scoring Pairs

**Figure 2:**
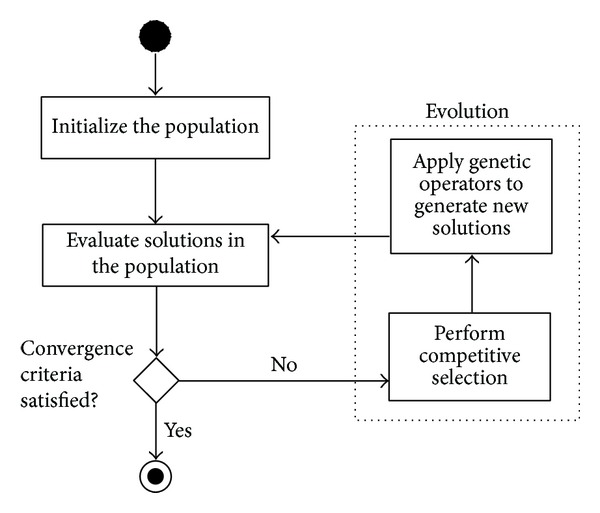
A general framework of evolutionary algorithm.

In this section, we would like to propose EvoTSP—an evolutionary algorithm for top-scoring pairs. Evolutionary algorithms [18] belong to a family of metaheuristic methods which represent techniques for solving a wide variety of difficult optimization problems. The general framework of EA (see Figure 2) is inspired by biological mechanisms of evolution. The algorithm operates on a population of individuals and each individual represents a candidate solution to the target problem. Individuals are assessed using a quality measure named the fitness function which measures their performance and those with higher fitness are usually more often selected for reproduction. Genetic operators such as mutation and crossover modify new generations of individuals, producing new offspring. This guided random search (offspring usually inherits some traits from its ancestors) is stopped when some convergence criteria are satisfied.

### 4.1. Representation and Initialization

Each individual is represented in its actual form as a potential solution. It is composed of a group of *k* top-scoring pairs similarly to *k*-TSP. As there are no restrictions on disjoint gene pairs, the EvoTSP is able to represent the TST solution with the 2 top-scoring pairs that involve only three genes. In the analogous way, TSN, *k*-TSP, or even variations of *k*-TSN can be represented in EvoTSP.

**Figure 3:**
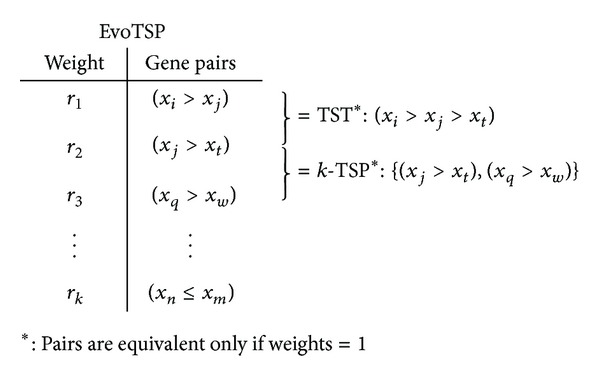
An example representation of EvoTSP model.

In this paper, we also propose additional parameter *r* for each pair of genes that represents its weight. This way, some gene pairs have higher influence than others on the final decision. This idea is completely new in TSP as aforementioned algorithms used a simple majority voting where each top-scoring pair's vote has the same weight. The purpose of using unweighted voting in TSP and all its extensions was probably directed by the necessity of limiting computational requirements.  Figure 3 shows an example EvoTSP model, which includes possible representation of *k*-TSP and the TST solution.

We could generate initial population randomly to cover the entire range of possible solutions; however, due to the large solution space, we decided to speed up evolutionary search and seed initial population with good solutions (default number of individuals in population equals 100).

Each initial individual has a random number of gene pairs (0 < *k* ≤ 5) created with the mixed dipole strategy [19] and constructed as follows. Among feature vectors located in the node two objects from different classes are randomly chosen. Next, an effective top-scoring pair is constructed with 2 randomly selected genes. By the effective top-scoring pair, we understand the pair of genes which separates two objects from different classes. In other words, genes *i* and *j* can constitute effective top-scoring pair only if there are at least two instances *q* and *w* that are from different classes and one of the relations is satisfied:

(4) $$\begin{array}{c} x_{iq}>x_{jq},\;\;x_{iw}\leq x_{jw}, \end{array}$$

or the opposite:

(5) $$\begin{array}{c} x_{iq}\leq x_{jq},\;\;x_{iw}>x_{jw}. \end{array}$$

This operation is repeated until *k* pairs is selected. All created gene pairs have equal weights (parameter *r*_*i*_ = 1 where *i* = 1,…, *k*). With this strategy we are able to limit the number of initial individuals which select only one class.

### 4.2. Fitness Function

Fitness function is one of the most important and sensitive elements in the design of the evolutionary algorithm. It drives the evolutionary search process by measuring how good a single individual is in terms of meeting the problem objective. Direct minimization of prediction error measured on the learning set usually results in overfitting and leads to spurious results.

In case of EvoTSP, we need to balance the error of classification and the number of genes that build the classifier. We have applied a similar idea that was used in the cost complexity pruning in the CART system [20]. The fitness function is maximized and has the following form:

(6) $$\begin{array}{c} \text{Fitness}=Q_{\text{Reclass}}-\alpha *\left(2*k+u\right), \end{array}$$

where *Q*_Reclass_ is the reclassification quality on the training set, *k* is the number of gene pairs, and *u* is the number of unique genes in top-scoring pairs that were used to build the classifier. The *α* parameter is the relative importance of the complexity term specified by user (default value is 0.005). Penalty associated with the classifier complexity increases proportionally with the number of genes that constitute the top-pairs. To reduce overfitting and to encourage searching relation between more than two genes, unique genes are doubly penalized. It should be noticed that there is no optimal value of *α* for all possible datasets and tuning it may improve classifier results for specific problem. Further research on setting this parameter automatically on a particular training data is planned.

### 4.3. Genetic Operators

To maintain genetic diversity, two specialized genetic operators corresponding to the classical cross-over and mutation were applied. Each evolutionary iteration starts with selecting individuals from the population that will be affected by the genetic operators. Probability of applying a cross-over operator equals 0.5 for each individual. With the same probability a mutation operator can also be applied. Next, one of the variants of genetic operator is selected.

We propose two variants of recombination:

- a randomly chosen pair of genes is exchanged between two affected individuals. Probability of pairs to exchange equals 0.9;
- a randomly chosen pair from the best individual founded so far replaces a random pair from the affected individual. In this variant only one individual is modified and the probability of this variant equals 0.1.

If the mutation operator is chosen, one of the variants with equal probability of being drawn is applied to the individual:

- add a new pair of genes created with the mixed dipole strategy;
- remove randomly chosen pair;
- replace randomly chosen pair by the new one created with the mixed dipole strategy;
- exchange one feature from randomly chosen pair;
- increase/decrease the weight of the randomly chosen pair (by multiplying or dividing by 2);
- switch the relation sign among randomly chosen pair.

### 4.4. Selection and Termination Condition

Ranking linear selection [18] is applied as a selection mechanism. In each iteration, a single individual with the highest value of fitness function in current population is copied to the next one* (elitist strategy)*. In addition, this strategy is partially boosted by possible cross-over of individuals from current population with the best individual founded so far. Evolution terminates when fitness of the best individual in the population does not improve during fixed number of generations (default value: 1000). In case of a slow convergence, maximum number of generations is also specified (default value: 10000), which allows us to limit the computation time.

## 5. Results and Discussions

In this section, all performed experiments are presented. At first, we share some details about datasets and settings of tested algorithms. Next, we validate and discuss the overall performance of EvoTSP solution and its competitors with respect to classification accuracy and its size.

### 5.1. Datasets and Setup

Performance of classifiers was investigated on several public available microarray datasets deposited in NCBI's Gene Expression Omnibus [21] and summarized in Table 1. All datasets are binary classification problems and mainly refer to the studies of human cancer. As the data was not predivided we used typical 10-fold cross-validation as it was the only option in AUREA software [16].

We confront EvoTSP with three competitors: the primary solution TSP and its two main extensions: *k*-TSP and TST (TSN with *N* = 3). To obtain comparison results, we used the AUREA software, which is an open-source system for identification of relative expression molecular signatures [16]. Classification was performed with default parameters for all algorithms through all datasets and to ensure stable results average score of 20 runs is shown. A statistical analysis of all obtained results was performed with the Friedman test and the corresponding Dunn's multiple comparison test (significance level equal to 0.05) as recommended by Demšar [22].

The AUREA software sets the maximum number of top-scoring pairs (parameter *k*) for *k*-TSP to 10 by default. In addition, all algorithms except EvoTSP operate on a subset of genes for analysis based on the differential expression of the presented gene set (the Wilcoxon signed-rank test was used to choose the most differentially expressed genes between the defined classes). Authors [16] state that this feature selection step have dramatic effect on the computational complexity of the algorithms and by limiting the set of genes, problem of over-fitting can be mitigated. In case of EvoTSP we have decided not to use any feature selection and allow searching for relations through all high and low-ranked genes.

### 5.2. Comparison of Top-Scoring Family Algorithms Methods

Table 2 summaries classification performance for the proposed solution EvoTSP and its competitors: TSP, TST, and *k*-TSP. The model size of TSP and TST is not shown as it is fixed and equals correspondingly 2 and 3. We had to use approximation of *k*-TSP size as AUREA software did not allow checking the *k* value during cross-validation; therefore, the value of *k* on full dataset treated as a training set is presented.

Results show that, in general, the existing extensions, TST and *k*-TST, outperform TSP in terms of accuracy. The price for better performance is the higher complexity of the classification model, which for *k*-TSP is 5.75 times higher (an average value from 8 datasets) than TSP size and almost 4 times than TST. Slightly larger size of classification model is not a problem, as all tested algorithms are simple to analyze; however, checking several different genes per model may be considered difficult in biological interpretation, which is the case for *k*-TSP.

In the last two columns of Table 2 we present the results of the proposed solution. We can observe that the accuracy of the classifier in 6 out of 8 datasets is the highest. However, for the last two databases EvoTSP accuracy score is slightly lower than *k*-TSP. Additional experiments showed that the convergence of EA in EvoTSP is too slow for that particular set. When the maximum number of generation in EA was increased, the proposed algorithm managed to have similar or even outperform *k*-TSP on both datasets.

According to the Friedman test, there is a statistically significant difference (*P* value of 0.0003) in the accuracy of all versions. Based on Dunn's Multiple Comparison Test Difference, there is a statistically significant difference in classification quality between EvoTSP, TSP, and the TST algorithm. Although there were no statistical differences in accuracy between EvoTSP and *k*-TSP, there is one in the size of their models. The size of classification model of proposed solution remains small, in contrast to *k*-TSP, making the EvoTSP a good tool for identifying gene-gene interactions with direct clinical applicability. In Table 2 we can also observe that the standard deviation of accuracy for solutions was on similar level.

Total time to build an EvoTSP model varies between 1 and 8 minutes on a typical PC (Intel Core I5, 4 GB RAM), depending on the dataset and it is few times longer than for AUREA software which was from tens of seconds to a minute. However, it should be noted that EvoTSP works without any feature selection which is a must for AUREA software (checking of all combinations of pairs would take many orders of magnitude more).

## 6. Conclusion

In this paper, we propose the EvoTSP system for solving classification problems using microarray data. Our approach is a hybrid solution that combines the power of EA and relative expression algorithms. We have designed several variants of specialized operators to mutate and cross-over individuals and a fitness function that helps mitigating the overfitting problem. With the new weighted gene pairs voting and extended representation of top-scoring pairs that involve different variants of TSP, we were able to significantly improve TSP accuracy with still relatively small size of classification model. Application of EAs allows exploring much larger solution space and searching for different, more complex relations between genes.

In this paper we only focus on the general concept of EvoTSP as an effective tool; therefore, we do not enclose any biological aspects of the rules generated by proposed system or case studies on particular datasets. Furthermore improvement is still required especially in terms of fitness functions to handle cost-sensitive and multiclass problems. Speeding up the convergence of the EA is also desirable and can be achieved by application of local optimizations (memetic algorithms), new specialized operators, and self-adaptive parameters. Finally, more work on preprocessing datasets, gene selection, and using additional problem-specific knowledge is also required to improve EvoTSP classification accuracy and rule discovery.

## Figures and Tables

**Table 1:** Details of tested gene expression datasets.

Datasets	Number of features	Number of instances
GDS2771	22215	192
GSE10072	22284	107
GSE17920	54676	130
GSE19804	54613	120
GSE25837	18631	93
GSE27272	24526	183
GSE3365	22284	127
GSE6613	22284	105

**Table 2:** Comparison of top-scoring algorithms, including accuracy with its standard deviation and the number of unique genes that build classifier's model.

Datasets	TSP	TST	*k*-TSP		EvoTSP	
	Accuracy	Accuracy	Accuracy	Size	Accuracy	Size
GDS2771	57.2 ± 2.4	61.9 ± 2.8	62.9 ± 3.3	10	65.6 ± 2.0	4.0
GSE10072	88.7 ± 2.6	89.4 ± 2.1	90.1 ± 2.5	6	96.5 ± 1.3	2.1
GSE17920	64.9 ± 3.5	63.7 ± 4.7	67.2 ± 3.2	10	78.1 ± 2.6	2.8
GSE19804	93.5 ± 1.7	92.8 ± 1.5	94.1 ± 1.6	10	96.2 ± 1.1	2.1
GSE25837	56.0 ± 4.0	60.5 ± 5.1	58.4 ± 4.0	14	66.9 ± 5.6	3.1
GSE27272	47.3 ± 4.8	50.1 ± 3.8	56.2 ± 2.2	18	66.2 ± 1.1	2.7
GSE3365	81.9 ± 2.6	84.2 ± 2.7	87.2 ± 2.1	14	86.1 ± 2.8	4.1
GSE6613	49.5 ± 3.5	51.7 ± 2.8	55.8 ± 5.3	10	53.6 ± 5.4	6.1

Average	67.4 ± 3.3	69.3 ± 3.2	71.5 ± 3.0	11.5	76.2 ± 2.7	3.4
